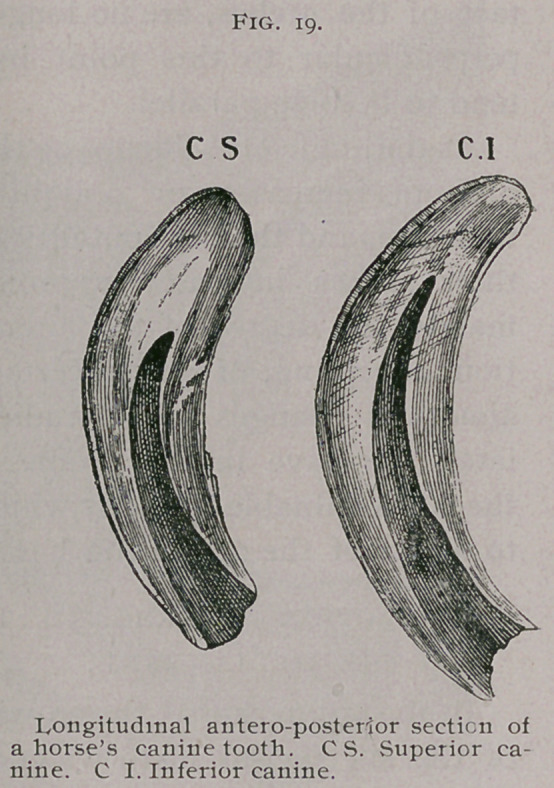# Age of the Horse, Ox, Dog, and Other Domestic Animals

**Published:** 1890-04

**Authors:** R. S. Huidekoper

**Affiliations:** Veterinarian


### AGE OF THE HORSE, OX, DOG, AND OTHER DOMESTICATED ANIMALS.

By R. S. Huidekoper, M.D., Veterinarian.

[*Continued from page 172.*]

*d. The Table of the Incisors.*

The table of the incisors is the free portion or crown of these teeth, which becomes worn by friction with the hard substances which the animal takes as food, and by the constant contact with the teeth of the opposite jaw. After the high anterior and lower posterior borders of the virgin incisors have been worn away, the table is established. At first this consists, on each tooth, of an oblong plate, wider from side to side, which is surrounded by the layer of cement directly covering the peripheral layer of enamel; inside of this is found the zone of yellowish and softer dentine, usually somewhat depressed on account of its lesser resistance to friction; in the middle, the central enamel or border of the cup surrounds the variable quantity of cement which may be deposited in it. (Fig. 9, Fig. 10, A, Fig. 14.)

Fig. 15.

Scheme of sections of wedge-shaped incisor.

As the table gradually encroaches upon the wedge-shaped tooth (Fig. 15), it becomes proportionately narrower in its transverse diameter, and as it becomes oval in shape the cup is found nearer the posterior border of the tooth instead of in the centre. The dental star, or dark-colored dentine which takes the place of the papilla, now appears between the cup and the anterior border of the tooth (Fig. 10, B). A still further destruction from use brings the table to the middle of the original tooth, when it assumes the round form, the cup of central enamel has disappeared, and the dental star is now midway between the anterior and posterior borders. (Fig. 10, C.)

Still further use brings the triangular form, and finally the biangular ; in these two forms the dental star gradually becomes larger and, at times, when the papilla has not been entirely replaced by the dentine, a cavity is found which, by the uninformed, has been mistaken for the cup. (Fig. 10, C and D.) Figure 15 shows the forms at the various parts of the original tooth.

*e. The Direction of the Incisors.*

The direction of the incisors, or the position which they hold in regard to the jaw, is to be studied, first, in profile, considering the relative angle which the incisors of the upper and lower jaw

FIG. 16.

Arch of the Incisors uncovered, to show their successive planes of contact. [Goubaux and Barrier].

have to each other; and second, in face, considering the position which they hold in regard to the median line.

1. DIRECTION OF THE PLANE OF CONTACT OF THE TWO JAWS. (FIG. 16).

In a young horse the incisors meet and form an arch which, if viewed in profile, represents the half of a circle, so that a tangent drawn from the point of contact of the two jaws is perpendicular to their tables. But as the progressive wearing of the table brings it nearer to the roots of the teeth, the half circle changes to the form of an ogive, which becomes more and more acute as the surface of contact, which is displaced above and below parallel to itself, extends gradually from the primitive diameter. Consequently the tangents, *aa'*, *bb'*, *cc'*, drawn from the new points (*a*, *b*, *c*) of contact of the arches, are no longer perpendicular to this point, but tend to become parallel.

Fig. 17.

Incisors of the two jaws seen by their anterior face to show their direction in regard to the median line. [Goubaux and Barrier.]

As the angle of incidence of the incisors increases in obliquity with age, and the horizontality of their arches indicates approximately the degree of the alteration, excepting, of course, certain abnormal changes to be studied later, we have in the profile of the jaw a valuable factor by which to judge of the age of the horse.

### 2. DIRECTION IN REGARD TO THE MEDIAN LINE.

In the young horse, the crowns of the six incisor teeth widened from side to side, while their roots are flattened in their transverse diameter, cause the teeth to take a position on the end of the jaw like the ribs of an open fan. They diverge from the alveolar cavities, in which they are imbedded in the bone, toward the circumference formed by their crowns. But, as age advances, the crowns become worn off and the teeth are pushed farther and farther out of their cavities. The roots, which were at first almost in contact with each other, gradually separate and widen, while the circumference constantly diminishes as the teeth become worn down, until they assume a parallel position, and finally converge, instead of diverging, at their free extremities. The intermediate teeth become separated from the pincers on the one side, and the corner teeth become separated from them on the other, until a distinct space is visible between them, which is filled by a pale gum.

*En resumé* in regard to the general direction of the incisors:

1. The incidence of the arches acquires a greater obliquity with age.

FIG. 18.

Upper right-hand canine tooth of a horse. A. External face. B. Internal face.

FIG. 19.

Longitudinal antero-posterior section of a horse's canine tooth. C S. Superior canine. C I. Inferior canine.

2. Their incurvation and their transverse diameter diminish.

3. The teeth, at first diverging from their roots, become parallel and finally converge toward their free extremities.

THE TUSHES, TUSKS, CANINE TEETH,

The tushes are four in number in the adult horse; they are rudimentary or absent in the mare. In certain sections of the country superstition has attributed sterility to mares provided with them, which is, however, absolutely unfounded.

The superior tusks are placed one on each side of the upper interdental spaces at the point of union of the superior maxillary and intermaxillary bones. The lower ones are placed on either side of the maxilla always closer to the incisor teeth than those of the upper jaw, so that they are invariably in front of the latter.

The canine teeth are curved in their long axis with the concavity backward and they project slightly outward. The external face is covered with small longitudinal parallel striations. The internal face has a conical eminence pointing toward the free extremity of the tooth and separated from its borders by a deep gutter. The tusks have a pulp cavity relatively much larger than that of the incisors, which, however, also becomes filled with a discolored dentine as the tooth advances in age.

### HISTOLOGY.

The tusks are composed of ivory or dentine, inclosing a pulp cavity and surrounded with enamel.

There are no temporary tusks in the horse, although small rudimentary spicula sometimes are found before the permanent teeth appear.

[TO BE CONTINUED.

## Figures and Tables

**Fig. 15. f1:**
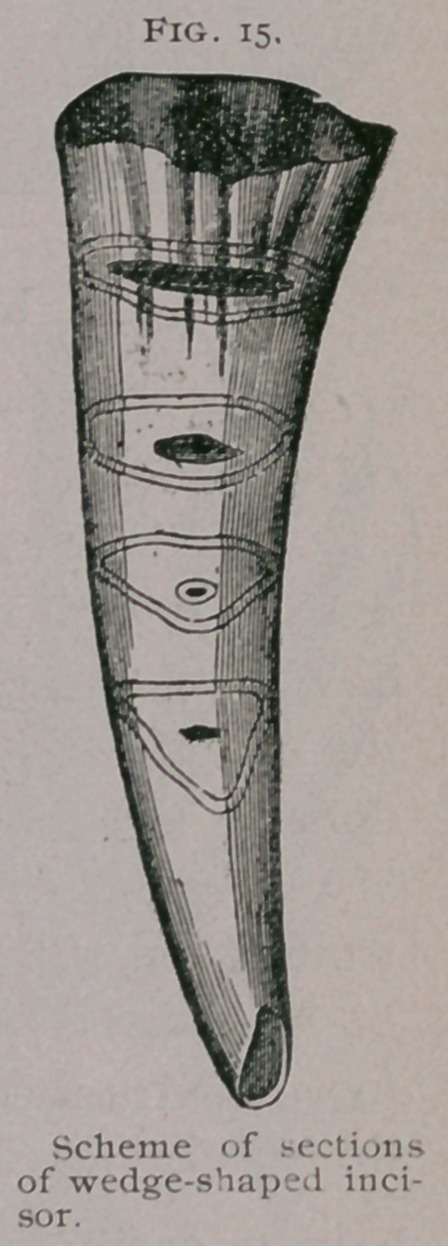


**Fig. 16. f2:**
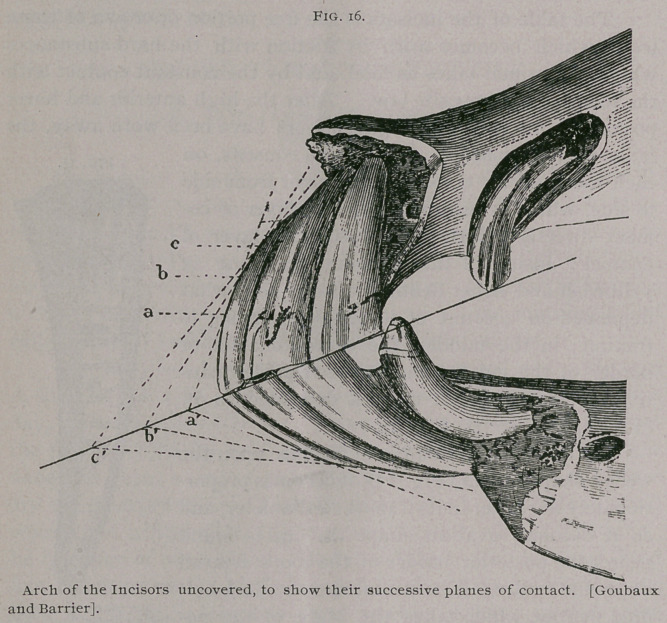


**Fig. 17. f3:**
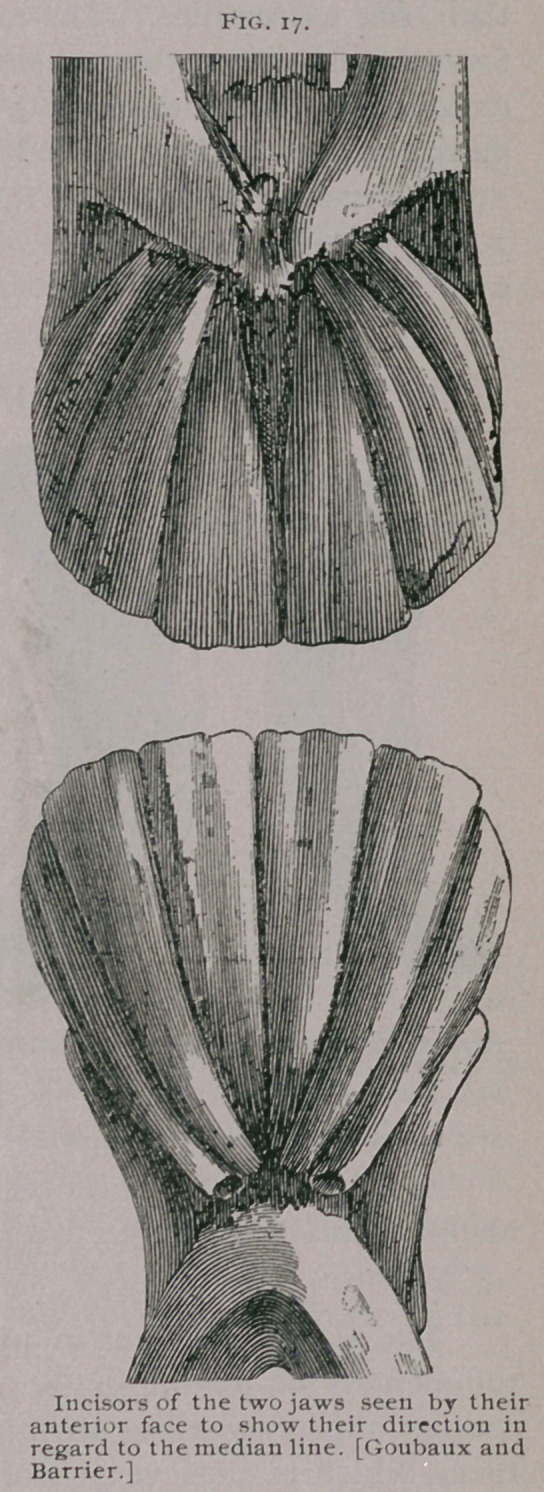


**Fig. 18. f4:**
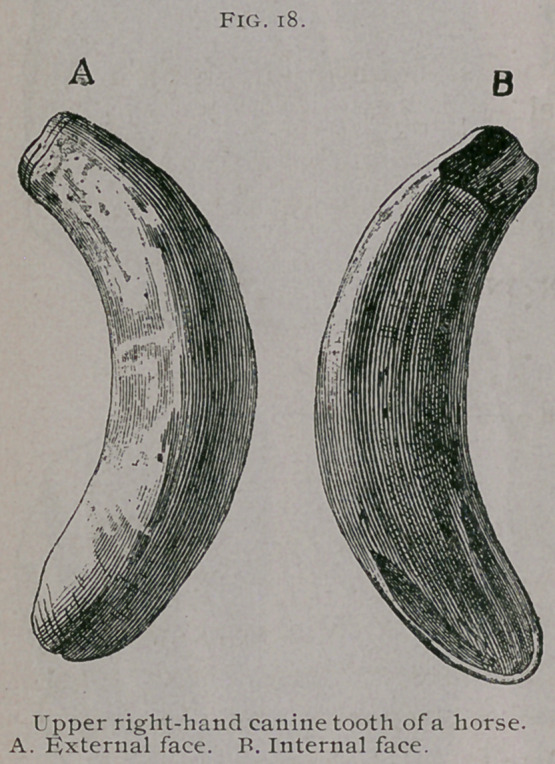


**Fig. 19. f5:**